# Motivations of Human Helping Behavior towards Dogs

**DOI:** 10.3390/vetsci9030145

**Published:** 2022-03-21

**Authors:** Serenella d’Ingeo, Gabriele Ferlisi, Michele Minunno, Giovanni L. Palmisano, Gianluca Ventriglia, Marcello Siniscalchi, Angelo Quaranta

**Affiliations:** 1 Animal Physiology and Behavior Unit, Department of Veterinary Medicine, University of Bari Aldo Moro, 70121 Bari, Italy; serenella.dingeo@uniba.it (S.d.); m.minunno79@libero.it (M.M.); gianluca.ventriglia@uniba.it (G.V.); marcello.siniscalchi@uniba.it (M.S.); 2 Tree of Life Psychological Centre, 70124 Bari, Italy; gabriele.ferlisi@libero.it; 3 Centro Ricerche Sullo Stress Interpersonale (CRISI), Cooperativa Sociale ONLUS, 70125 Bari, Italy; gianlucapalmisano@yahoo.it

**Keywords:** dog, welfare, human–animal relationship, volunteers, cognitive therapy interventions

## Abstract

Human–dog interactions have a positive effect on human sociality and health. The relationship with dogs helps humans to cope with stress during an emotionally challenging period, such as the COVID-19 pandemic. During this period, a growing global interest in pets has been registered, including the volunteering for shelter/stray dog protection. However, a considerable increase of human dysfunctional interventions toward dogs has been observed in Southern Italy. In this study, we investigated the psychological characteristics of humans volunteering at animal shelter or engaged in stray dog protection. The effect of psychological training and education about dog ethological needs on volunteers’ helping behavior was also analyzed. We report that the intervention can improve volunteers’ physiological features and, consequently, may enhance human management and dog welfare.

## 1. Introduction

The reality of volunteering with animals in Italy is very complex. All citizens sharing a “deep love towards animals” could provide care for stray or shelter animals, both privately and through volunteering in animal protection organizations. Given this fluid and poorly structured reality, the inventory and the management of the volunteers acting in the field is particularly troublesome. The organization of training programs is difficult as well. Poor education poses the concrete risk of a scarce effectiveness of the interventions for the conservation of the species and the welfare of the animals concerned. Indeed, a growing number of dysfunctional interventions (e.g., the capture of non-socialized dogs or superficial/not suitable adoptions) have been registered by professionals working in the canine field in Southern Italy, particularly in the Apulia region. These actions are based on human helping behavior that finds its roots in the deep and lasting relationship with the dogs. Dogs (*Canis familiaris*) have been part of human society for longer than any other domestic species [1]. The unique relationship they entertain with humans, which has been recently demonstrated also for cats [2], could be related to the ability of these species to functionally recognize human emotions [3,4,5,6,7,8], which regulate their daily interactions. The close link between dogs and humans has been described as attachment [9], which is characterized by emotional closeness, knowledge of the other, the ability to provide assistance and protection, and/or the use of the other subject as a source of safety and comfort [10]. The relationship with dogs offers several benefits for humans. Dogs have positive effects on human health: they increase elderly people happiness, alertness, and reactivity, as well as producing benefits for people suffering from cardiovascular diseases and depression [11,12]. Dogs also influence the emotional and social aspects of human life. They can act as catalysts for social interactions [13,14] and could offer emotional support, particularly in times of emotional stress. Owners, indeed, are more likely to turn to their dogs than to friends or family [15]. They provide security and empathy, love, comfort, trust, and protection and may help humans to increase their self-esteem and decrease the sense of loneliness [16]. Dogs promote the sense of being necessary for another individual that depends on their care and protection [17]. 

Indeed, the relationship and interactions with dogs have been crucial to buffer and reduce the negative psychological impact of COVID-19 during the last two years [18,19,20,21,22]. Dogs have offered social support by physical contact with the owners [23] and an effective distraction from unsettling feelings during the pandemic [24], as well as a valuable motivation to engage in shared activities [25]. Most importantly, the interaction with animals has reduced or minimized the feeling of loneliness due to social distance measures [22,26]. The awareness of the positive effect of the interaction with animals, and in particular dogs, drove humans to look for dog support under the stress-prone conditions caused by COVID-19. Indeed, it has been registered a significant increase in the global interest on pet adoption (up to 250%) in the early phase of the pandemic [27]. This constitutes an unequivocal and clear sign of the profound value of human–dog relationship and the role of dogs in human society, particularly in Western cultures.

The high value people attribute to their relationship with animals, which sometimes is experienced as an integral part of human life, could be explained by the psychological theory of helping behavior [28]. Rogers defined the helping relationship as “a relationship in which at least one of the two protagonists has the purpose of promoting growth, development, maturity and the achievement of a more adequate way of acting in the other. […] In other words, a helping relationship could be defined as a situation in which one of the participants tries to favor, in one or both parties, a greater appreciation of the subject’s personal resources and a greater possibility of expression” [29]. It has been reported that humans help when feeling emotionally attached and empathetic towards someone [30]. Human helping behavior toward animals aims at protecting and providing care for them [31], leading humans to invest their resources, including time and money.

Several studies have been carried out to investigate the relevant motivations besides human helping behavior. It could be driven by altruistic concerns, which are based on others (i.e., helping others according to human own civil responsibility); biocentric or ecocentric concerns, which are based on living beings, ecosystems, and the environment; or selfish motivations, which are based on the ego (e.g., for improving own skills or relieving guilt) [32,33]. The interactions with animals, indeed, improve human interpersonal relationships by decreasing loneliness and isolation, particularly in elderly and anxious people [33,34]. The helping behavior includes the care and the assistance of others (also animals), which could relieve personal suffering and promote social consideration [35]. However, it could potentially lead to dysfunctional relationships as has been described for animal hoarding disorders or for dog aggression toward humans [36,37]. 

The altruistic actions toward animals can be also related to empathic feeling as a sincere motivation to help, which aims at producing benefits for others (empathy–altruism model [38]). However, the emotional component of empathy could be evoked from personal distress and could therefore be the trigger for helping behavior, solely on the basis of a selfish motivation: the observation of others suffering causes an increase of tension in the observer, resulting in the helping behavior as a discomfort relief.

The identification of the motivations inducing the helping behavior towards others (including animals) is complex, since it is influenced by different factors and situations. However, it is fundamental for the understanding and the improvement of the quality of the aid. If the motivation of the help is mainly oriented to fulfill self-needs, it could inhibit the real understanding of others’ needs, and could invalidates the help. It is possible that apparent altruism may have underlying selfish motivations. Di Michele and colleagues [39] showed that humans with a low self-esteem in stressful situations would help others to dampen their discomfort, but only when no other solution is possible (they would prefer the flight response).

Professionals of the canine field in the Apulia region (in the Southern Italy), including university professors, researchers, veterinarians, dog educators and instructors, veterinary behaviorists, and kennel managers, observed that most of the regional volunteers display dysfunctional helping behaviors that are not entirely oriented towards dog needs (e.g., the capture of non-socialized dogs or superficial/not suitable adoptions). They also show a certain degree of resistance to change their perspective and acquire a new one. We therefore wondered if there are some common psychological characteristics in the people involved in stray dog/shelter volunteering that could underlie the increasing number of dysfunctional interventions registered in the Apulia region. This could serve as a starting point to set up professional and educational training for volunteers “working” in shelters and for free roaming dog protection. The awareness of the motivation underling the helping behavior as well as the knowledge of dog ethological needs (i.e., dog needs to perform natural behaviors) may significantly improve human management and dog welfare.

## 2. Materials and Methods

### 2.1. Subjects

Participants were volunteers at animal shelters or engaged in stray dog protection. They were recruited through social media from Apulian animal shelter organizations and students of the University of Bari. Subjects were assigned to the experimental or control groups using a matched pairs design, wherein pairs of participants were matched in terms of age and gender. One member of each pair was then placed into the experimental group and the other member into the control group. A total of 122 volunteers participated in the study. The experimental group included 71 subjects, 51 women and 20 men, with a mean age of 38.41 years (S.D. = 11.433), whereas the control group included 51 subjects, 36 women and 15 men, with a mean age of 38.18 (S.D. = 13.48). The two groups did not statistically differ in terms of age (t (1120) = 0.103; *p* > 0.05) and gender (χ^2^ = 0.881; *p* > 0.05).

### 2.2. Questionnaires

Questionnaires were administered to the experimental and control groups to evaluate volunteers’ psychological characteristics. In particular, the Italian version of the Young Schema Questionnaire (YSQ [40]), the Idea Inventory questionnaire [41], and the Italian version of the Acceptance and Action Questionnaire II (AAQ-II [42]) were used.

1. The Young Schema Questionnaire (YSQ-L3) [40] was employed to verify the presence and the frequency of 18 early maladaptive schemas in the analyzed population. According to Young, a maladaptive schema is a concept or a model formed by memories, emotions, thoughts, and somatic sensations, developed in childhood or adolescence, present in all phases of life, and has little functionality [40]. The activation of such schemas (i.e., abandonment, distrust/abuse, emotional inhibition, inadequacy/shame, social exclusion/alienation, dependence/incompetence, failure, vulnerability to danger or disease, entanglement/underdeveloped self, claims/grandiosity, insufficient self-control or self-discipline, submission, seeking approval or recognition, self-sacrifice, negativity/pessimism, emotional inhibition, strict standards/hypercriticism, punishment) depends on the presence of subjects’ unmet needs. Their activation impairs the subjects’ ability of understanding others’ real needs. The YSQ-L3 is a self-report questionnaire, made up of 232 items and based on the Schema Therapy model [40]. Each participant is asked to rate each statement on a 6-point Likert scale ranging from 1 (“it is completely untrue for me”) to 6 (“it describes me perfectly”) [40]. The YSQ-L3 has demonstrated satisfactory test–retest reliability and internal consistency, as well as convergent and discriminant validity [43]. Regarding the internal consistency of the Italian version of the YSQ-L3, Cronbach’s alpha for all the schemas showed moderate to high values, ranging, respectively, from 0.804 to 0.916 in the clinical sample and from 0.895 to 0.916 in the non-clinical sample [44]. The convergent validity of the Italian version of YSQ-L3 was investigated by using Pearson’s r correlation coefficients with well-known depression and anxiety measures (TFI, Teate Depression Inventory; STICSA, State–Trait Inventory for Cognitive and Somatic Anxiety). Almost all the schemas positively and significantly correlated with these two clinical measures [44]. 

2. The Idea Inventory was used to evaluate the presence of irrational thoughts and beliefs. It is an effective tool for the assessment of subjects’ attitudes towards key aspects of human life as knowledge, desires, choices, ways of evaluating and judging oneself, others, and their environment. The Idea Inventory [41] is a 33 item Likert scale (3 point scale, ranging from 1 = agree, 2 = uncertain, to 3 = disagree), which assess the 11 irrational beliefs of Ellis’s Rational Emotional Therapy conceptualization (REBT). The total scale score is obtained by summing the scores of the 33 items (range 33–99). It showed satisfactory test–retest reliability coefficients (product–moment) and group change over time (Pearson’s r > 0.80) [41,45]. Moreover, it had good convergent validity (Pearson’s r correlation with the general measure of psychopathology (Minnesota Multiphasic Personality Inventory, MMPI)). Therefore, the higher the scores at the Idea Inventory, the higher the probability to show psychological symptoms (MMPI) [46]. For the purpose of this study, the total scale was employed to perform the statistical analysis.

3. The Italian version of the Acceptance and Action Questionnaire II version (AAQ-II [42,47]) is a self-report measure that was designed to assess experiential avoidance and inflexibility. According to the Acceptance and Commitment Therapy (ACT [48]), the experiential avoidance is a construct featured by subjects’ reluctance to remain in contact with painful thoughts and feelings. It leads subjects to act for avoiding and/or changing the “negative” thoughts and feelings [47]. Psychological inflexibility refers instead to a “rigid dominance of psychological reactions over chosen values and contingencies in guiding actions” [47]. It has been found that its presence is negatively related to subjects’ quality of life, perceived health, and positive emotional experiences [48]. The AAQ-II is a one-dimensional measure of avoidance/psychological inflexibility, consisting of a 10 item Likert-type scale ranging from 1 (“never true”) to 7 (“always true”). The total score is calculated by summing the scores obtained from the 10 items (range 10–70). High scores reflect high levels of psychological inflexibility and experiential avoidance as well as low levels of general acceptance. Regarding psychometric characteristics, AAQ-II showed satisfactory reliability for group comparisons [49]. Moreover, the mean alpha coefficient of the AAQ-II was 0.84 (range 0.78–0.88), demonstrating satisfactory internal consistency. Furthermore test–retest reliability, determined by performing the Cronbach’s alpha at 3 and 12 months, displayed good to acceptable values (0.81 and 0.79, respectively) [49]. The Italian adaptation used in the present study showed high internal consistency (Cronbach’s alpha = 0.83). However, test–retest reliability over a 12 month period was modest (Cronbach’s alpha = 0.61). Regarding the concurrent validity (obtained performing Pearson’s r), high scores of AAQ-II are significantly related to depression, anxiety, and alexithymia, as well as to psychological issues [48].

### 2.3. Procedures

**Figure 1:**
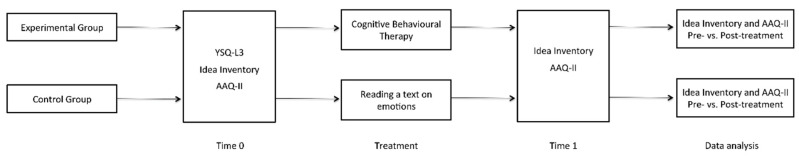
Schematic representation of the study procedure.

The experimental and control groups received to two different treatments. The experimental group (GT) underwent a group cognitive-behavioral therapy whereas the control group (GC) was involved in the reading of a text regarding the primary and secondary emotions (Figure 1). Specifically, the cognitive-behavioral therapy consisted of 4 h sessions that were structured as follows: 

- 1 h: a psycho-educational intervention to describe mental functioning, in order to promote greater awareness in terms of beliefs, think, emotions, and behavior as well as a greater awareness of the motivations behind the helping behavior (personal needs vs. altruism).

- 2 h: Rational Emotional Therapy (REBT [49]) aiming at increasing psychological flexibility and reducing dysfunctional attitudes. The REBT is a cognitive behavioral psychological treatment developed by Albert Ellis. It is based on the assumption that human emotional reactions and behavioral responses depend mainly on the way in which individuals interpret and think about reality. According to this theoretical framework, irrational thoughts and beliefs cause the expression of dysfunctional behaviors, emotional distress, and mental suffering. The REBT allows humans to identify dysfunctional beliefs and act to refute them through a set of cognitive techniques. This results in a change of dysfunctional beliefs into functional beliefs, thus promoting psychological well-being and the acceptance of themselves and others. Specifically, in this study, human irrational thoughts and beliefs were firstly described by the psychologist and then refuted through a group dispute guided by the same therapist.

- 1 h: the description of dog ethological needs by veterinary behaviorists through video support. 

The intervention consisted of three sessions for both groups, with an inter-session interval of 15 days. The Idea Inventory and the AAQ-II questionnaires were submitted to each group before the beginning of the first session (time 0) and at the end of the last session (time 1). This provided evidence about the effect of the treatment on subjects’ irrational thoughts/beliefs and psychological inflexibility/experiential avoidance. In addition, the YSQ-L3 was administered at time 0 to assess the potential presence of activated maladaptive schemas (that could not be modified by the treatment adopted) and its relationship with the irrational thoughts/beliefs and psychological inflexibility/experiential avoidance of the analyzed population. Participants were instructed to complete the questionnaires considering their feelings on their emotional levels. No time limit was set for completing the questionnaires for both groups. 

### 2.4. Data Analysis

The Kolmogorov–Smirnov and the Levene test were used to analyze the data and the homoskedasticity of the distributions (i.e., the property that a collection of random variables has when they all have the same variance), respectively. The chi-squared test was employed to evaluate the differences between the experimental and control group in the frequency of the active schemas measured by the YSQ-L3. According to the validated interpretation of the test, the score attributed to each maladaptive schema expresses the activation level as very high, high, medium, and low. Therefore, considering the scores reported, for each subject, the activation level (very high, high, medium, and low) of each of the maladaptive schemas described by the YSQ-L3 was obtained. 

**Figure 2:**
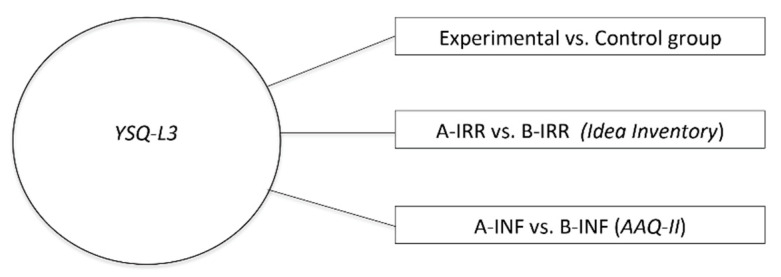
Schematic representation of the data analysis.

To study the general characteristics of the sample population, we examined the relationship between the maladaptive schemas (measured by YSQ-L3) and the irrational thoughts/beliefs (measured by Idea Inventory) and psychological inflexibility/experiential avoidance (measured by AAQ-II). We divided the whole sample of participants (experimental and control group, *n* = 122) into two subsamples according to their response to the Idea Inventory and AAQ-II by using the median-split method. For each questionnaire, the median of the continuous distribution of scores was used as a cut-off to divide the population into two categories: high and low scores. In particular, for the Idea Inventory, the score median value used as a cut-off was 70. The sample was therefore divided into two subsamples: high irrationality group (A-IRR: score > 70) and low irrationality group (B-IRR: score < 70). Regarding the AAQ-II, the score median value used as a cut-off was 30. The sample was therefore divided into high inflexibility group (A-INF: score > 30) and low inflexibility group (B-INF: score < 30). The chi-squared test was used to evaluate the differences between the high vs. low irrationality group and between the high vs. low inflexibility group for each of the maladaptive schemas described by the YSQ-L3 (Figure 2).

The median split is considered to be a robust procedure exempt from the risk of rising misleading results (e.g., Type 1 errors) when there are no multicollinearity problems [50]. We used the variance inflation factor (VIF) to identify multicollinearity. As reported in the statistic literature, VIF values higher than 10.0 are often index of multicollinearity [50]. We obtained VIF values ranging from 1.199 and 3.514, thus below both the aforementioned value of 10.0 and the most conservative cut-off of 4.0 [51]. 

Finally, Student’s t-test for paired dependent samples was used to evaluate the differences in the scores of the Idea Inventory and the AAQ-II between pre- and post-treatment in the experimental and control groups. All the participants were informed about the aims of the intervention, and written informed consent was obtained from all of them. 

## 3. Results

### 3.1. Activated Schemes

The chi-squared test revealed that, with the exception of the submission scheme, the experimental and control groups did not statistically differ in terms of activated schemas. In particular, regarding the submission scheme, the control group obtained a higher frequency of high-level active schemas than the experimental group (χ^2^ = 9.498, *p* < 0.05; see Table S1). We can therefore state that, except for the above-mentioned schema, the two groups were essentially comparable for the variables age, gender (see Section 2.1), and active schemas.

Considering the total sample of volunteers (*n* = 122), the very high and high activation of the self-sacrifice schema in both groups deserves further consideration. Specifically, 24 subjects activated this schema at a very high level and 43 subjects at a high level. Specific mention is also made for the following schemas: strict standards/hypercriticism (16 subjects: very high activation; 25 subjects: high activation); abandonment (13 subjects: very high activation; 15 subjects: high activation); claims/grandiosity (11 subjects: very high activation; 16 subjects: high activation); and punishment (7 subjects: very high activation; 12 subjects: high activation). 

### 3.2. Relationship between the Activated Schemes and Irrational thoughts and Beliefs

Data analysis showed that maladaptive schemas (assessed by the YSQ-L3) were more activated in the high irrationality group (A-IRR) than in the low irrationality group (B-IRR). In particular, statistically significant differences were found in the activation of the schemas: abandonment (χ^2^ = 22.64, *p* < 0.001), distrust/abuse (χ^2^ = 14.293, *p* < 0.005), social exclusion/alienation (χ^2^ = 14.583, *p* < 0.005), failure (χ^2^ = 14.962, *p* < 0.005), vulnerability to disease (χ^2^ = 16.067, *p* < 0.001), entanglement/underdeveloped self (χ^2^ = 15.313, *p* < 0.005), submission (χ^2^ = 10.34, *p* < 0.05), self-sacrifice (χ^2^ = 10.885, *p* < 0.05), emotional inhibition (χ^2^ = 17.458, *p* < 0.001), strict standards/hypercriticism (χ^2^ = 10.17, *p* < 0.05), claims/grandiosity (χ^2^ = 30.586, *p* < 0.0001), insufficient self-control/self-discipline (χ^2^ = 14.774, *p* < 0.005), search for approval/acknowledgment (χ^2^ = 19.752, *p* < 0.0001), negativity/pessimism (χ^2^ = 21.741, *p* < 0.0001), and punishment (χ^2^ = 18.954, *p* < 0.0001) (see Table S2). 

### 3.3. Relationship between the Activated Schemes and Psychological Inflexibility

Results showed that maladaptive schemas (assessed by the YSQ-L3) were more activated in the high-inflexibility group (A-INF) than in the low-inflexibility group (B-INF) (assessed by the Acceptance and Action Questionnaire, AAQ-II). In particular, statistically significant differences were found in the activation of the following schemas: emotional deprivation (χ^2^ = 16.686, *p* < 0.001), abandonment (χ^2^ = 21.626, *p* < 0.001), distrust/abuse (χ^2^ = 11.176, *p* < 0.05), social exclusion/alienation (χ^2^ = 16.074, *p* < 0.001), failure (χ^2^ = 9.481, *p* < 0.001), vulnerability to disease (χ^2^ = 8.08, *p* < 0.05), entanglement/underdeveloped self (χ^2^ = 10.461, *p* < 0.05), submission (χ^2^ = 9.546, *p* < 0.05), emotional inhibition (χ^2^ = 24.792, *p* < 0.0001), claims/grandiosity (χ^2^ = 12.804, *p* < 0.005), insufficient self-control/self-discipline (χ^2^ = 12.703, *p* < 0.005), search for approval/acknowledgment (χ^2^ = 8.236, *p* < 0.05), and negativity/pessimism (χ^2^ = 18.928, *p* < 0.0001). No significant differences were found for the activated schemes: dependence/incompetence, self-sacrifice, strict standards/hypercriticism, and punishment (*p* > 0.05) (see Table S3).

### 3.4. The Effect of the REBT-Intervention

A statistically significant difference in the scores of the AAQ-II and Idea Inventory was observed between pre- and post-treatment in the experimental group (AAQ-II: t = −14.47, *p* < 0.0001; Idea Inventory: t = 3.7, *p* < 0.0001). On the contrary, no statistically significant differences in the scores of the two tests between the pre- and post-intervention were found for the control group (*p* > 0.05) (see Table S4). 

## 4. Discussion

The present study aimed at investigating the potential presence of some common psychological characteristics in people involved in stray dog/shelter volunteering that may underlie the increasing number of dysfunctional interventions registered in the Apulia region. Specifically, we analyzed the activation of maladaptive schemas (described by Young [40], see paragraph 2.2) and its relationship with the presence of psychological inflexibility and irrational thoughts/beliefs. We found a high activation of self-sacrifice (71 subjects, 58.20%), strict standards/hypercriticism (41 subjects, 33.61%), abandonment (28 subjects, 22.95%), pretensions/grandiosity (27 subjects, 22.13%), and distrust/abuse (22 subjects, 19%) schemas in the total population. Considering that abandonment, pretensions/grandiosity, and distrust/abuse are classified as unconditional or primary schemas (whereas self-sacrifice and strict standards/hypercriticism are conditioned or secondary) [43], their activation provide interesting insight into the motivations behind volunteers’ behavior toward dogs. Broadly speaking, the activation of schemas determines how a person relates to others during social interactions. The activation of the abandonment schema, which reflects the subject’s abandonment issues, could increase the need for physical closeness and lead to the expression of overprotective behaviors. In these subjects, the fear of being abandoned is reflected onto the animals, thus making the need to protect them from danger and loneliness becomes of primary importance. In the region analyzed in this project, dysfunctional rescue of puppies or lonely stray dogs occurs frequently, on the base of an overestimation of the dangers the animals are exposed to (causing the dogs to be deprived of freedom and familiar social relationship). The activation of distrust/abuse schema instead, which reflects the subject’s fear of being deceived, could cause difficulties in interacting and collaborating with colleagues and professionals (including veterinarians) on the management of animals and may therefore impact the dog welfare. Finally, the activation of the pretensions/grandiosity schema, which reflects the feeling of being superior, could cause the use of animals as a means to exert power and control (e.g., by acting for the rescue of a considerable number of animals). We found that the activation of the above-mentioned schemas is also related to the presence of psychological inflexibility and irrational thoughts/beliefs in the analyzed population. This result is consistent with previous studies reporting that early maladaptive schemas are associated with both high level of inflexibility and irrational beliefs and thoughts [52,53,54,55]. They suggest that a strong activation of maladaptive schemas (frustrated needs) could affect subjects’ cognitive appraisal of events, promoting the onset of dysfunctional beliefs and the use of dysfunctional problem-solving strategies in an attempt to avoid negative thoughts and emotions. Taken together, the activation of maladaptive schemas and the presence of psychological inflexibility and irrational thoughts/beliefs could suggest the nature of the dysfunctional interventions registered in the analyzed territory since they directly impact the quality of volunteers’ activity toward dogs. One of the most relevant examples of dysfunctional interventions concerns the capture of feral and semi-feral dogs that live in family groups in balance with the environment. The lack of socialization both with humans and the urban environment causes the expression of severe behavioral problems (i.e., anxiety, phobia, and aggression) that considerably impair the animal adoption rate and generally lead to long-term sheltering (even for the animal’s entire life). Superficial or not suitable adoptions constitute another typical example of dysfunctional interventions. They might cause severe disorders in the human–dog relationship that could expose owners to the concrete risk of aggression.

The presence of activated maladaptive schemas, irrational thoughts and beliefs, and psychological inflexibility has a considerable importance when referring to the effect of the COVID-19 pandemic on human–dog relationships [19]. The government restrictions, as well as social isolation, work loss, and mourning, became relevant stressors that could have impacted people’s vulnerability to stressful life situations, which has been related to the activation of schemas [56]. The maladaptive schemas from different domains were indeed positively associated with COVID-19 anxiety and psychological distress [56]. The affection between human and dogs is well known as an effective way to reduce stress during stress-prone conditions and to help in buffering depression and anxiety in the context of social isolation [19,20,23,57]. Indeed, an increased in human willingness to engage in interaction with pets in the early phase of COVID-19 pandemic was noted [27]. However, during this period, a dramatic increase of dysfunctional interventions was observed in the Apulia region by the professionals of the canine field. It could be possible, therefore, that the increase of human stress levels during the COVID-19 pandemic could have led people to engage more often in dysfunctional helping behavior toward dogs with severe consequences for their welfare. 

Interestingly, we found significant differences in psychological inflexibility and irrational thoughts and beliefs in the experimental group after the REBT treatment, as previously reported [58]. It suggests that a proper training and education of volunteers could be effective for reducing the rate of dysfunctional interventions. Therefore, our study highlights the need for a proper volunteer education program that, together with a training program for dogs to facilitate a proper adaptation to the family environment [59] and the refinement of breeding and adoption processes [60], could significantly impact the welfare of rescued dogs. Although in Italy there are reporting obligations under the Italian Law 201/2010 [61] regarding the education of shelter volunteers, they are fully disregarded. Our findings further corroborate the effectiveness of training programs for shelter volunteers reported in the recent literature [62,63,64,65]. It has indeed been shown that training programs for environmental enrichment and dog walking procedures (e.g., the standard protocol “Safewalk” [65]) as well as behaviorally based dog training improve dog welfare. Moreover, they increase the adoption rate and decrease the length of stay in shelters [63,64]. 

To our knowledge, our study suggests for the first time that a cognitive therapy intervention may improve the effectiveness of volunteers’ helping behavior toward dogs since it promotes a greater awareness of the motivations and emotions underlying volunteering. It could also potentially affect volunteers’ quality of life and relationship with others, and, most importantly, it could improve human actions for the safeguarding of animal welfare.

This study highlights some points that could be improved in future steps. First of all, the sample here examined may not be representative of the volunteer category since only people mostly inclined to receive training in psychology have joined the study. Secondly, we did not use a control sample that perfectly matched the number of the experimental group. It was related to the high mortality resulting from a low motivation to adhere to the reading intervention. However, the sample did not statistically differ for socio-demographic variables (e.g., age and gender) and for the early maladaptive schemes activated, psychological inflexibility, and beliefs that were dysfunctional/irrational at baseline. In addition, we only used self-report measures that could represent a noteworthy limitation for the purposes of this pilot study. Finally, it was not possible to carry out a follow-up to evaluate the permanence and stability of the results obtained over time. Despite these limitations, one of the major strengths of this research is that, to our knowledge, our study suggests for the first time the presence of early maladaptive schemas in the sample population. This result would not serve for a classification of the sample population as clinical, but it rather highlights the effectiveness of the REBT in reducing the volunteers’ psychological inflexibility and irrational beliefs/thoughts. This could significantly improve the quality of human helping behavior toward dogs. Future studies could analyze the potential differences of the psychological features here considered between the sample and the general population of the region.

## 5. Conclusions

Our study suggests that volunteers’ psychological features should be taken into account since the potential activation of early maladaptive schemas could affect the understanding of the dogs’ needs and, consequently, the effectiveness of human interventions. Moreover, training as well as treatment courses based on REBT might be useful for the education of volunteers in order to reduce dysfunctional beliefs and psychological inflexibility, and, therefore, improve individual psychological well-being and the effectiveness of their activities. On the other hand, the educational courses held by veterinary behaviorists would increase the knowledge of dog behavior and ethological needs, in order to improve the efficacy of volunteers’ intervention for protecting animal welfare. Further studies on larger samples would be useful in order to generalize the results obtained and to standardize a widespread intervention in the Italian volunteering panorama.

## Data Availability

Not applicable.

## Supplementary Materials

The following supporting information can be downloaded at: https://www.mdpi.com/article/10.3390/vetsci9030145/s1, Table S1: Differences in the frequencies of activated schemes in the experimental group (GT) and control group (GC); Table S2: Differences in the frequencies of activated schemes in the group with high level of irrational beliefs (A-IRR) and low level of irrational beliefs (B-IRR); Table S3: Differences in the frequencies of activated schemes in the high and low inflexibility group; Table S4: Scores of AAQ-II, Idea Inventory pre- and post treatment.
