# Supplementary material for: Motivations of Human Helping Behavior towards Dogs
Source: Vet Sci. 2022 Mar 21;9(3):145. doi: 10.3390/vetsci9030145 (PMC8953770; doi:10.3390/vetsci9030145)
Supplement: Supplementary file 1 [file vetsci-09-00145-s001.zip › vetsci-1617405-supplementary.pdf]

Supplementary Information

Title of Manuscript:

Motivations of Human Helping Behavior towards Dogs

Serenella d'Ingeo[1§], Gabriele Ferlisi[2§], Michele Minunno[1], Giovanni L. Palmisano[3], Gianluca Ventriglia[1], Marcello Siniscalchi[1], Angelo Quaranta[1]

[1] Animal Physiology and Behavior Unit, Department of Veterinary Medicine, University of Bari Aldo Moro, 70121 Bari, Italy; serenella.dingeo@uniba.it (S.d.); m.minunno79@libero.it (M.M.); marcello.siniscalchi@uniba.it (M.S.); gianluca.ventriglia@uniba.it (G.V.)

[2] Tree of Life Psychological Centre; 70124 Bari, Italy; gabriele.ferlisi@libero.it (G.F.)

[3] Centro Ricerche sullo Stress Interpersonale (CRISI), cooperativa sociale ONLUS; 70125 Bari, Italy; gianlucapalmisano@yahoo.it (G.P.)

§ These authors contributed equally to this work as first authors.

* Correspondence: angelo.quaranta@uniba.it (A.Q.); Tel.: +39-080-544-3927

Table S1. Differences in the frequencies of activated schemes in the experimental group (GT) and control group (GC). YSQ-L3: Young Schema Questionnaire; f: frequency; n.s.: not significant (p>0.05). Chi-squared test of Pearson

Activated schemes YSQ-L3		GT (N = 71)	GC (N = 51)	χ^2	*df*	*p*
		f	f			
Emotional Deprivation	Very High	45	34	2.992	3	n.s.
	High	8	2			
	Medium	10	6			
	Low	8	9			
Abandonment	Very High	7	6	0,615	3	n.s.
	High	9	6			
	Medium	7	7			
	Low	48	32			
Distrust/Abuse	Very High	4	8	4,132	3	n.s.
	High	7	3			
	Medium	20	11			
	Low	40	29			
Social exclusion/Alienation	Very High	3	1	0,723	3	n.s.
	High	3	3			
	Medium	11	9			
	Low	54	38			
Inadequacy/Shame	Very High	1	0	0,941	3	n.s.
	High	1	1			
	Medium	3	3			
	Low	66	47			
Failure	Very High	0	2	4,078	3	n.s.
	High	4	5			
	Medium	4	4			
	Low	63	40			
Dependence/Incompetence	Very High	0	1	2,027	3	n.s.
	High	1	1			
	Medium	7	3			
	Low	63	46			
Vulnerability to disease	Very High	1	2	0,985	3	n.s.
	High	3	2			
	Medium	12	10			
	Low	55	37			
Entanglement/Underdeveloped self	Very High	0	2	4,391	3	n.s.
	High	5	1			
	Medium	12	8			
	Low	54	40			
Submission	Very High	2	1	9,498	3	< .05
	High	0	6			
	Medium	6	6			
	Low	63	38			
Self-sacrifice	Very High	16	8	3,759	3	n.s.
	High	26	17			
	Medium	12	16			
	Low	17	10			
Emotional inhibition	Very High	2	4	3,122	3	n.s.
	High	6	5			
	Medium	8	9			
	Low	55	33			
Strict Standards /Hypercriticism	Very High	8	8	0,732	3	n.s.
	High	14	11			
	Medium	17	12			
	Low	32	20			
Claims/Grandiosity	Very High	4	7	5,389	3	n.s.
	High	8	8			
	Medium	18	6			
	Low	41	30			
Insufficient Self-control/self-discipline	Very High	8	2	5,592	3	n.s.
	High	2	6			
	Medium	11	8			
	Low	41	30			
Search for Approval/ Acknowledgment	Very High	2	1	4,829	3	n.s.
	High	2	1			
	Medium	3	8			
	Low	64	41			
Negativity/Pessimism	Very High	2	3	2,501	3	n.s.
	High	7	4			
	Medium	7	9			
	Low	55	35			
Punishment	Very High	4	3	3,719	3	n.s.
	High	10	2			
	Medium	16	15			
	Low	41	31			

Table S2. Differences in the frequencie of activated schemes in the group with high level of irrational beliefs (A-IRR) and low level of irrational beliefs (B-IRR). YSQ-L3: Young Schema Questionnaire; f: frequency; n.s.: not significant (p>0.05). Chi-squared test of Pearson.

Activated schemes YSQ-L3		A-IRR (N = 61)	B-IRR (N = 61)	χ^2	*df*	*p*
		f	f			
Emotional Deprivation	Very High	5	5	6,061	3	n.s.
	High	12	4			
	Medium	10	7			
	Low	34	45			
Abandonment	Very High	11	2	22,64	3	< .001
	High	13	2			
	Medium	9	5			
	Low	28	52			
Distrust/Abuse	Very High	10	2	14,293	3	< .005
	High	9	1			
	Medium	13	18			
	Low	29	40			
Social exclusion/Alienation	Very High	4	0	14,583	3	< .005
	High	6	0			
	Medium	13	7			
	Low	38	54			
Inadequacy/Shame	Very High	1	0	3,888	3	n.s.
	High	2	0			
	Medium	4	2			
	Low	54	59			
Failure	Very High	2	0	14,962	3	< .005
	High	7	2			
	Medium	8	0			
	Low	44	59			
Dependence/Incompetence	Very High	1	0	7,343	3	n.s.
	High	2	0			
	Medium	8	2			
	Low	50	59			
Vulnerability to disease	Very High	3	0	16,067	3	< .001
	High	5	0			
	Medium	16	6			
	Low	37	55			
Entanglement/Underdeveloped self	Very High	2	0	15,313	3	< .005
	High	5	1			
	Medium	16	4			
	Low	38	56			
Submission	Very High	3	0	10,34	3	< .05
	High	5	1			
	Medium	9	3			
	Low	44	57			
Self-sacrifice	Very High	30	13	10,885	3	<.05
	High	12	16			
	Medium	11	16			
	Low	8	16			
Emotional inhibition	Very High	5	1	17,458	3	<.001
	High	10	1			
	Medium	12	5			
	Low	34	54			
Strict Standards /Hypercriticism	Very High	13	3	10,17	3	< .05
	High	15	10			
	Medium	11	18			
	Low	22	30			
Claims/Grandiosity	Very High	11	0	30,586	3	<.0001
	High	15	1			
	Medium	7	17			
	Low	28	43			
Insufficient Self-control/self-discipline	Very High	9	1	14,774	3	<.005
	High	7	1			
	Medium	11	8			
	Low	34	51			
Search for Approval/ Acknowledgment	Very High	3	0	19,752	3	<.0001
	High	3	0			
	Medium	11	0			
	Low	44	61			
Negativity/Pessimism	Very High	5	0	21,741	3	<.0001
	High	10	1			
	Medium	12	4			
	Low	34	56			
Punishment	Very High	5	2	18,954	3	<.0001
	High	11	1			
	Medium	20	11			
	Low	25	47			

Table S3. Differences in the frequencies of activated schemes in the high and low inflexibility group. YSQ-L3: Young Schema Questionnaire; A-INF: high inflexibility group; B-INF: low inflexibility group; f: frequency. Chi-squared test of Pearson.

Activated schemes YSQ-L3		B-INF (N = 61)	A-INF (N = 62)	χ^2	*df*	*p*
		f	f			
Emotional Deprivation	Very High	1	9	16,686	3	< .001
	High	4	12			
	Medium	5	12			
	Low	48	31			
Abandonment	Very High	2	11	21,626	3	< .0001
	High	2	13			
	Medium	4	10			
	Low	50	30			
Distrust/Abuse	Very High	3	9	11,176	3	< .05
	High	1	9			
	Medium	14	17			
	Low	40	29			
Social exclusion/Alienation	Very High	1	3	16,074	3	< .001
	High	0	6			
	Medium	4	16			
	Low	53	39			
Inadequacy/Shame	Very High	0	1	5,393	3	< .145
	High	0	2			
	Medium	1	5			
	Low	57	56			
Failure	Very High	0	2	9,481	3	< .05
	High	2	7			
	Medium	1	7			
	Low	55	48			
Dependence/Incompetence	Very High	0	1	6,403	3	>.05
	High	0	2			
	Medium	2	8			
	Low	56	53			
Vulnerability to disease	Very High	0	3	8,08	3	< .05
	High	0	5			
	Medium	10	12			
	Low	48	44			
Entanglement/Underdeveloped self	Very High	0	2	10,461	3	< .05
	High	1	5			
	Medium	5	15			
	Low	52	42			
Submission	Very High	1	2	9,546	3	< .05
	High	0	6			
	Medium	3	9			
	Low	54	47			
Self-sacrifice	Very High	18	25	1,051	3	>.05
	High	14	14			
	Medium	13	14			
	Low	13	11			
Emotional inhibition	Very High	0	6	24,792	3	< .0001
	High	1	10			
	Medium	3	14			
	Low	54	34			
Strict Standards /Hypercriticism	Very High	6	10	1,687	3	>.05
	High	11	14			
	Medium	13	16			
	Low	28	24			
Claims/Grandiosity	Very High	1	10	12,804	3	< .005
	High	4	12			
	Medium	12	12			
	Low	41	30			
Insufficient Self-control/self-discipline	Very High	1	9	12,703	3	<.005
	High	2	6			
	Medium	6	13			
	Low	49	36			
Search for Approval/ Acknowledgment	Very High	0	3	8,236	3	<.05
	High	0	3			
	Medium	3	8			
	Low	55	50			
Negativity/Pessimism	Very High	0	5	18,928	3	<.0001
	High	0	11			
	Medium	6	10			
	Low	52	38			
Punishment	Very High	2	5	6,632	3	>.05
	High	5	7			
	Medium	10	21			
	Low	41	31			

Table S4. Scores of AAQ-II, Idea Inventory pre- and post treatment. M= mean; S.D.= Standard deviation. T-test for paired samples

	Pre-treatment		Post-treatment				
Variables (N=71)	M	S.D.	M	S.D.	t	df	p
Experimental Group (N=71)							
AAQ-II	28,8	9,585	26,37	9,011	-14,47	70	<.0001
Idea	69,35	11,084	81,94	10,44	3,7	70	<.0001
Control Group (N=51)							
AAQ-II	30,82	10,303	31,43	9,976	0,95	50	> .05
Idea	68,88	11,461	68,39	12,045	-1,97	50	>.05
